# 

**Published:** 1890-07-05

**Authors:** 


### NOTICE TO CORRESPONDENTS.

All MS., Letters, Books for Review, and other matters intended for the Editor should be addressed The Editor, The Lodge, Porchester Square, London, W.

The Editor cannot undertake to return rejected M.S., even when accompanied by stamped directed envelope.

**Notice to Advertisers and Subscribers.**

All Advertisements, Orders for Copies of The Hospital, and Business Communications should be addressed to The Publisher, *not to the Editor*, The Hospital, 140, Strand, W.C.

**Bound Volumes of "The Hospital."**

Vol. VI., containing full page portraits of T.R.H. the Prince and Princess of Wales, and Miss Florence Nightingale, is obtainable at 4s., post free.

Orders will be received for Vol. VII., 4s. post free. Cases for binding may be had of the Publisher, at 1s. 6d. each.

**To Residents, Secretaries, and Matrons.**

The Editor will be glad to have the Regulations and each Report as issued by Asylums, Hospitals, Convalescent and Nursing Institutions, as well as those of other Charities, and an early copy in duplicate of all Appeals and Festival Notices, etc., addressed to *The Editor, The Lodge, Porchester Square, London, W.* Any superintendent, secretary, matron, or other chief official who regularly sends such information may be registered, on application to the Publisher, to receive free of cost a cover for binding The Hospital in half-yearly volumes.

### BURDETT'S HOSPITAL ANNUAL FOR 1890

Is now ready, and may be obtained of the Publisher, The Hospital Offices, 140, Strand, W.C., price 2s. 6d. limp boards, or 3s. 6d. cloth. All orders must be accompanied by a remittance.

### Scraps and Gleanings.

Dr. Stuart Nairne figures in *The Bailie* for June 19th.

After a month in England, Dr. Lawrie is returning to Hyderabad.

Lady Wolseley will open Woolwich Cottage Hospital on July 26th.

Hospital Saturday in Lincoln brought in £131; £6 more than last year.

On Tuesday the Freemasons of North Connaught gave a banquet to Surgeon Parke.

Hospital Sunday Parade at Caversham resulted in a collection amounting to £45.

Mrs. Bishop has got a grant of land in Kashmir on which to erect a hospital to hold 60 beds.

Moreton-on-Marsh Cottage Hospital possesses the chair used by Charles I. on the scaffold.

A summer camp for Manchester lads has been pitched again this year. This is a true health-giving movement.

Archdeacon Burney lately preached in aid of Surbiton Cottage Hospital. The offertories amounted to £81.

Mr. T. Backhouse entertained the Hospital Saturday collectors of Sunderland at tea, and some interesting speeches were made.

The poor little Duke of Albany has commenced the arduous duties of royalty already; he helped at a bazaar at Sandown last week.

At the second annual meeting of the Cyprus Society, an appeal was made for funds to commence the Gordon Hospital at Kyrenia.

Nine deaths from sunstroke were recorded in St. Louis last week. Please St. Louis, we should like a little of your superfluous sun.

They have had an interesting patient at the Princess Alice Memorial Hospital: A man with several French nails driven deep into the top of his head.

The Empress Frederick has collected 500.000 marks (£25,000) towards the cost of the Children's Hospital at Berlin, of which the foundation-stone was laid in her presence on June 20th.

The Executive Council of the French Exhibition, West Brompton, will give a grand French fête on Saturday, July 5th, in aid of the New French Hospital, which opened on Thursday, July 3rd.

Admiral J. C. Prevost presided at the annual meeting of the Shipwrecked Fishermen and Mariners' Royal Benevolent Society. (What a fearful name!) Some 9,000 persons were assisted last year.

More big sums for the Hospital Sunday Fund; West London Synagogue, £249 7s. 6d.; St. Philip, Kensington, £102 3s. 11d.; St. Matthew, Bayswater, £227 13s.; St. Stephen, South Kensington, £233.

A new lifeboat of the Suffolk and Norfolk type was launched at Lowestoft, last week. The vessel, which is called the Stock Exchange, is the largest in the service, and is the 29th on the Norfolk and Suffolk coasts.

Sir Andrew Clark has rented Dalquharran Castle, the seat of Mr. Kennedy, Dunure, which is one of the finest and most beautiful places in Ayrshire, for the autumn months, and Mr. Gladstone will probably be his guest there in October for a few days.

A telegram from Pesth states that nine of the women who were arrested some time ago for poisoning their husbands have been put on their trial at Mitrovitza. One of the accused has confessed that she killed by arsenic her niece, her husband, and her father-in-law.

An inquiry instituted in Germany shows that there are still forty-nine German Waterloo veterans living. Five of them are in their 100th year, one in his 101st, one in his 105th, and one in his 107th year. The youngest was born in 1799, and the oldest is Johann Doyse, of Rehorst, in the province of Schleswig-Holstein.

Mrs. G. Anderson, M.D., read the list of the London School of Medicine for Women scholarships, prizes, and certificates to a large gathering of the students and their friends. She said that she was glad to see so many students had been successful in the study of forensic medicine, a somewhat unfeminine subject.

The Chairman and executive committee of the South London Polytechnic Institutes have resolved upon inviting sixteen architects to supply designs in competition for an institute in Battersea Park Road. The cost of the building is not to exceed £40,000, and a premium of £400 is offered for the design adjudged to be the best.

The leper question is at present exciting great attention in Bombay. There are in that city 1,000 lepers, most of them quite destitute. The Government gives no sign of any intention to proceed with legislation providing for them; and when they are sent to asylums the lepers refuse to remain, and the authorities have no power to detain them.

At the opening of the new branch hospital of the Seamen's Society, purses were presented by the following ladies: Mrs. Poulson, Mrs. Elmslie, Miss Alderton, Miss Batsford, Miss Chapman, Miss Lethbridge, Miss Moore, Miss Roberts (of New York), Miss Swabey (of Exeter), Miss Worthington. The Society will need new annual subscribers to carry out the work now begun.

The *British Medical Journal* understands that the Apothecaries' Society are considering the propriety of selling their old botanical garden, known as the "Physick Garden," at Chelsea, which is in the vicinity of the Chelsea Hospital. The freehold is said to be of the value of upwards of £30,000, and the maintenance involves a cost of £600 a-year; and it is thought the fund might be better applied.

The death is announced of Admiral John Ross Ward, who organized the working department of the National Lifeboat Institution, which, during his thirty-one years' tenure of office as General Inspector of Lifeboats, was the means of saving at least 20,000 lives. He retired in 1883, being then seventy years of age, and was presented with the gold medal of the institution for his invaluable services.

The new wing of the Middlesex Hospital, and the new additions to the Hospital Française, Leicester-square, have both been fitted with the Longford wire mattresses and bedsteads (Wood's patent). Some additional mattresses have also been supplied to St. John's Hospital, Leicester Square; the Western Hospital, Seagrave Road; Metropolitan Asylums Board; the New West Riding Asylum at Menston; and the County Asylum, Lancaster.

